# Iatrogenic ureteric injuries following abdomino-pelvic operations: a 10-year tertiary care hospital experience in Tanzania

**DOI:** 10.1186/s13017-015-0011-z

**Published:** 2015-03-12

**Authors:** Phillipo L Chalya, Anthony N Massinde, Albert Kihunrwa, Samson Simbila

**Affiliations:** Department of Surgery, Catholic University of Health and Allied Sciences-Bugando, Mwanza, Tanzania; Department of Obstetrics /Gynecology, Catholic University of Health and Allied Sciences-Bugando, Mwanza, Tanzania; Department of Urology, Catholic University of Health and Allied Sciences-Bugando, Mwanza, Tanzania

**Keywords:** Ureteric injuries, Iatrogenic, Abdomino-pelvic operations, Management, Outcome, Tanzania

## Abstract

**Background:**

Iatrogenic ureteric injuries are rare complications of abdomino-pelvic surgery but associated with high morbidity and even mortality. There is paucity of data regarding iatrogenic ureteric injuries in Tanzania and Bugando Medical Centre in particular. This study describes our experience in the management and outcome of ureteric injuries following abdomino-pelvic operations outlining the causes, clinical presentation and outcome of management of this condition in our local setting.

**Methods:**

This was a retrospective descriptive study of patients with iatrogenic ureteric injuries following abdomino-pelvic operations that were managed in Bugando Medical Centre between July 2004 and June 2014.

**Results:**

A total of 164 patients (M: F = 1: 1.6) were studied. Of these, 154 (93.9%) were referred to Bugando Medical Centre having had their initial surgeries performed at other hospitals, whereas 10 (6.1%) patients sustained ureteric injuries during abdomino-pelvic surgery at Bugando Medical Centre. The median age at presentation was 36 years. The most common cause of iatrogenic ureteric injuries was total abdominal hysterectomy occurring in 69.2% of cases. The distal ureter was more frequently injured in 75.6% of cases. Suture ligation was the commonest type of injury accounting for 36.6% of patients. One hundred and sixteen (70.7%) patients had delayed diagnosis but underwent immediate repair. Ureteroneocystostomy was the most frequent reconstructive surgery performed in 58.0% of cases. Of the 164 patients, 152 (92.7%) were treated successfully. Twelve (7.3%) patients died in hospital. The main predictors of deaths were delayed presentation, deranged renal function tests on admission, missed ureteric injuries and surgical site infections (P < 0.001). The overall median length of hospital stay was 12 days. Follow up of patients was generally poor as more than half of patients were lost to follow up.

**Conclusion:**

Total abdominal hysterectomy still accounts for most cases of iatrogenic ureteric injuries in our environment. Meticulous surgical technique as well as identification of the course of the ureter and associated anatomic locations where injury is most likely to occur is important to decrease the risk of ureteric injury. Timely recognition of ureteric injury and its management is associated with good outcome.

## Background

Injury to the ureter is one of the most serious complications of any abdominal or pelvic procedure whether from gynaecological, urological or general surgical disease and the medico-legal implication has always been a major area of concern [1]. These complications can result in high morbidity and even mortality for the patients, which can create anxiety and psychosocial concerns for the patients and their spouses [2]. The morbidity arising from ureteric injury includes increased hospital stay, secondary invasive interventions, reoperation, potential loss of renal function and deterioration patient’s quality of life [3].

The incidence of iatrogenic ureteric injuries varies from 0.5–10% in different studies [4-6]. It can occur following any abdomino-pelvic surgery. Obstetric and gynaecological surgery, vascular surgery, general surgical procedures especially colorectal surgery and urological procedures are commonly responsible for ureteric injuries [6]. Endo-urological procedures also account for many ureteric injuries [6,7]. Traditionally, gynaecological procedures have been reported to account for between 50 and 75% of iatrogenic ureteric injuries since the ureter lies very near the female reproductive organs throughout its course from the pelvic brim to the bladder [8-10]. While recent reports from developed countries have however indicated a change in the pattern of these iatrogenic injuries with urological endoscopy being the major source, recent literature from the sub-Saharan Africa is relatively sparse, with the few ones available also indicating a change in pattern however of a different variety with a high incidence of injuries arising from gynaecological causes [11-14]. Intra-operative injury to the ureter is possible not only during complicated surgical procedures but also during uncomplicated procedures [10]. Such complication can occur even in the hands of experienced surgeons [10,15].

The risk factors for iatrogenic ureteric injury include nature and indication of the abdominal or pelvic surgery, patient related factors such as: pelvic adhesions from previous surgeries, history of pelvic radiation, enlarged uterus, pelvic malignancy, pelvic endometriosis, and anatomical abnormalities [16,17]. Experience of the operating surgeon may also be an important risk factor [18,19].

During abdomino-pelvic surgery, ureteric injury may be in the form of crush injury by a clamp, inadvertent diathermy injury, suture ligation, transaction, resection of a segment of ureter, kinking of ureter, and devascularization of a segment of ureter due to extensive dissection close to ureter. Patient may develop urinary fistula with adjacent organ or end up with non-functioning kidney later on [19,20].

The most important determinant of outcome of ureteric injuries is the interval between the injury and repair: the longer the interval, the worse the outcome [20]. Prompt intraoperative identification and appropriate correction decrease morbidity and eliminate mortality. However, most cases of ureteric injuries are recognized late [6,7,16,17,20]. Patients may present with flank pain, fever, prolonged ileus, ascites, urinary incontinence, anuria and azotemia with 5% patients present very late with hydronephrosis and non-functioning kidney [16,17].

The management of iatrogenic ureteric injuries in our environment poses major challenges to urologist practicing in developing countries such as Tanzania where most ureteric injuries after abdomino-pelvic surgeries are diagnosed late postoperatively with fever, loin pain, per-vaginal urine leak, prolonged ileus, oliguria, anuria and uraemic symptoms [21,22]. Some patients may remain asymptomatic and present years later with hydro-nephrotic, non-functioning kidney on investigations [21]. Lack of advanced diagnostic and therapeutic facilities is the hallmark of the disease in these countries. In addition to late presentation and lack of advanced diagnostic and therapeutic facilities, the majority of abdomino-pelvic operations in developing countries are performed by general practitioners and junior doctors who may have limited experiences in performing major abdomino-pelvic operations as well as prompt intraoperative identification and appropriate correction of ureteric injury [19,21,22],

There is a paucity of information regarding iatrogenic ureteric injuries in Tanzania and Bugando Medical Centre in particular. This is partly due to a paucity of published local data regarding this condition in this region. This study was designed to describe our experiences in the management and outcome of iatrogenic ureteric injuries, highlighting the causes, clinical presentation and treatment outcome of iatrogenic ureteric injuries in our local setting.

## Methods

### Study design and setting

This was a retrospective descriptive study of consecutive patients with iatrogenic ureteric injuries who were referred and managed in the department of urology of Bugando Medical Centre over a period of ten years from July 2004 to June 2014. Bugando Medical Centre is a tertiary and teaching hospital for the Catholic University of Health and Allied Sciences-Bugando (CUHAS-Bugando). It is located in Mwanza city and has a bed capacity of 1000.

### Study population

The study included all patients who were referred to the urology department with a suspected diagnosis of iatrogenic ureteric injury. Patients with incomplete data were excluded from the study. The information was obtained from medical record database and from patients’ files, operating theatre, surgical, urological and gynecological ward registries. Variables such as gender, age at the time of injury and side of injury were recorded as well as information about the indication for surgery, type of surgery performed, location of injury, when the injury was identified, method of repair and outcome of repair. The outcome of repair included post operative complications, length of hospital stay and mortality. A repair was deemed successful if there are no anastomotic related complications and the subsequent preservation of unilateral kidney function on follow up. Intravenous urography (IVU) was used to assess renal function and exclude strictures or hydronephrosis.

### Definition of terms

Iatrogenic ureteric injury was defined as any inadvertent injury to the ureter which necessitates additional period of observation or intervention. The recognition time of injury was arbitrarily divided into: intraoperative, early (within seven days of the primary operation) and late (after seven day of primary operation). The primary disease referred to the disease condition that necessitated the abdomino-pelvic surgery (primary procedure).

### Statistical data analysis

The statistical analysis was performed using the Statistical Package for Social Sciences (SPSS) version 17.0 for Windows (SPSS, Chicago, Illinois, United States). The median (and IQR) and ranges were calculated for continuous variables, whereas proportions and frequency tables were used to summarize categorical variables. The chi-square (*χ*2) test was used to test for the significance of association between the independent (predictor) and dependent (outcome) variables in the categorical variables. The level of significance was considered as P < 0.05. Multivariate logistic regression analysis was used to determine predictor variables that predicted the outcome.

### Ethical consideration

Ethical approval to conduct the study was obtained from the CUHAS-Bugando/BMC joint institutional ethic review committee before the commencement of the study.

## Results

Out of 6424 patients who had abdomino-pelvic operations performed either at our centre or in the peripheral hospital and referred here for management of complications of abdomino-pelvic operations during the study period, 176 (2.7%) had iatrogenic ureteric injuries. Of these, 12 patients were excluded from the study due to incomplete data. Thus, a total of 164 patients were enrolled in the study. One hundred and fifty-four (93.9%) patients with ureteric injuries following abdomino-pelvic surgery were referred to Bugando Medical Centre having had their initial surgeries performed at other hospital, whereas only 10 (6.1%) patients sustained ureteric injuries during abdomino-pelvic surgery at Bugando Medical Centre. The age of patients at presentation ranged from 24 to 74 years with a median age of 36 years (interquartile range, 34 to 38 months). The peak age incidence was in the age group 31–40 years. Out of 164 patients, 62(37.8%) were males and 102(62.2%) were females with a male to female ratio of 1: 1.6. The most common cause of iatrogenic ureteric injuries was obstetric and gynaecological procedures (55.5%) mainly total abdominal hysterectomy occurring in 69.2% of cases (Table 1). Gross pelvic adhesions from previous surgeries were reported in 6 (3.7%) patients. Previous history of pelvic inflammatory diseases was reported in 10 (6.1%) patients. Massive intraoperative bleeding during pelvic tumour surgeries was reported in 4 (2.4%) patients. No patient in this study had history of pelvic radiation or anatomical abnormalities. All patients who were referred from peripheral hospitals had their abdomino-pelvic surgeries performed by general practitioners who might have limited experience.

**Table 1:** **Etiology of iatrogenic ureteric injuries**

**Etiology of iatrogenic ureteric injuries**	**Frequency**	**Percentages**
**Gynaecological/obstetric operations**	**91**	**55.5**
• Total abdominal hysterectomy	63	69.2
• Caesarean section	14	15.4
• Excision of huge tubo-ovarian mass	12	13.2
• Vaginal operations	2	2.2
**General surgical operations**	**68**	**41.5**
• Large bowel resection (colectomy)	32	47.1
• Intraabdominal tumour excision	30	44.1
• Abdomino-perineal resection (APR)	4	5.9
• Pull through operation	2	2.9
**Urological operations**	**5**	**3.0**
• Endourological procedures	3	60.0
• Other urological operations	2	40.0

Out of 164 abdomino-pelvic surgeries, 72(43.9%) were elective and 92(56.1%) were emergency. Ninety-eighty (59.8%) patients had left sided ureteric injuries and (36.6%) had right sided ureteric injuries with a right-to-left ratio of 2.1: 1. Six (20.4%) patients had bilateral ureteric injuries. The distal ureter was more frequently injured in 75.6% of cases. Suture ligation was the commonest type of injury accounting for 36.6% of patients (Table 2). The diagnosis was made intra-operatively in 28 (17.1%) patients and it was characterized by urinary extravasations into the peritoneal cavity in 20 (12.2%) patients. In the remaining 136 (82.9%) patients the diagnosis was postoperative. Of these, the diagnosis was made early within seven days in 20(12.2%) and in 116 (70.7%) patients the diagnosis was made late after 7 days. The time interval from injury to urological consultations ranged from 2 to 172 days with a median of 14 days ((interquartile range, 12 to 16 days). Missed ureteric injuries were reported in 8 (4.9%) patients who presented with prolonged ileus, fever, frank pain, anuria, haematuria and elevated serum creatinine. Of these, 6 (75.5%) were found to have non-functioning kidney. The presenting features are shown in Table 3.

**Table 2:** **Demographic data and injury characteristics**

**Study variable**	**Frequency**	**Percentages**
Age		
• <40	71	43.3
• ≥40	93	56.7
Sex		
• Male	62	37.8
• Female	102	62.2
Nature of the primary surgery		
• Elective	72	43.9
• Emergency	92	56.1
Laterality		
• Left	98	59.8
• Right	60	36.6
• Bilateral	6	3.6
Site of injury		
• Upper (Proximal) third	12	7.3
• Middle third	28	17.1
• Lower (Distal) third	124	75.6
Type of injury		
• Suture ligation	60	36.6
• Ureteric transection	44	26.8
• Excision of ureteric segment	28	17.1
• Crush injury	16	9.8
• Uretero-vaginal fistula	12	7.3

**Table 3:** **Postoperative clinical presentation (N = 136)**

**Clinical presentation**	**Frequency**	**Percentages**
Leakage of urine per vagina or through the surgical wound	57	41.9
Loin pain/mass	30	22.1
Fever	23	16.9
Urinary incontinence	18	13.2
Abdominal pain	16	11.8
Haematuria	13	9.6
Peritonitism	10	7.4
Non-functioning kidney	9	6.6
Anuria	6	4.4

Pre-operative assessment included Ultrasound scan, Intravenous urography (IVU), Computed tomography (CT) scan, examination under anaesthesia, dye test and cystoscopy as indicated.

All patients except two who died before definitive surgery had surgical intervention. All the injuries were repaired as soon as possible after diagnosis was made and stabilization of their condition done. Ureteroneocystostomy was the most frequent reconstructive surgery performed in 58.0% of cases (Table 4).

**Table 4:** **Surgical procedures for the Treatment of Ureteric Injuries (n = 162)**

**Surgical procedures**	**Frequency**	**Percentages**
Ureteroneocystostomy	94	58.0
End to end uretero-ureterostomy	34	21.0
Psoas hitch + Ureteroneocystostomy	15	9.3
Psoas hitch + Boari flap + Ureteroneocystostomy	12	7.4
Nephrectomy	9	5.6
Percutanaous nephrostomy	7	4.3

Note: All patients with percutaneous nephrostomy underwent definitive surgery after stabilization of their condition.

A total of thirty-eight patients postoperative complications were recorded in 30 (18.5%) patients, the commonest being surgical site infections in 36.8% of patients (Table 5). A total of six patients required re-laparotomy for postoperative complications as follows; three patients for intra-abdominal abscess /peritonitis, two patients for wound dehiscence and one patient intra-abdominal bleeding after excision of huge intraabdominal tumor. One patient required ureteroscpy for postoperative ureteral stenosis. Other postoperative complications were treated successfully with good results.

**Table 5:** **Postoperative complications**

**Postoperative complications**	**Frequency**	**Percentages**
Surgical site infection	14	36.8
Postoperative pyrexia	6	15.8
Urinary tract infections	3	7.9
Intraabdominal abscesses/peritonitis	3	7.9
Wound dehiscence	2	5.3
Intra-abdominal haemorrhage	2	5.3
Paralytic ileus	1	2.6
Ureteral stenosis	1	2.6

The overall length of hospital stay (LOS) ranged from 1 day to 75 days with a median of 12 days. The LOS for non-survivors ranged from 1 day to 14 days (median 4 days). According to multivariate logistic regression analysis, patients who developed post-operative complications stayed longer in the hospital and this was significant (P = 0.011). Delayed diagnosis and treatment were also associated with longer hospital stay (P < 0.001).

Of the 164 patients, 152 (92.7%) were treated successfully and discharged home and the remaining twelve (7.3%) patients died in hospital. The causes of deaths were sepsis in six patients, renal failure in three patients and in the remaining patients the causes of death were not documented. According to multivariate logistic regression analysis, delayed presentation [OR = 3.8, 95% CI (2.4- 6.5), p = 0.011], deranged renal function tests on admission [OR = 2.9, 95%CI (1.7-8.6), p = 0.001], missed ureteric injuries [OR = 8.0, 95%CI (3.7-9.3), p = 0.000], and presence of postoperative complications mainly surgical site infections [OR = 5.3, 95% CI (3.5-11.5), p = 0.001] were the main predictors of mortality.

Patients were followed up for a period ranging between two and twelve months. At the end of follow up period only 65 (42.8%) of survivors were available for follow up and the remaining 87 (57.2%) were lost to follow up. Follow up evaluation mainly consisted of clinical evaluation and abdomino-pelvic ultrasound scan with IVU and cystoscopy when indicated.

## Discussion

Iatrogenic Injury to the ureter is the most common complication of abdomino- pelvic surgery, ranging from less than 1 to 10 percent of procedures, depending on the complexity of the procedure [4-6]. In this study, iatrogenic ureteric injuries were reported in 2.7% of all abdomino-pelvic operations, a figure which is comparable with what is reported in literature [4-6]. However, the figure of 2.7% in this study may actually be an underestimate and the magnitude of the problem may not be apparent because many cases may have been missed or excluded from the study due to missing data owning to the retrospective nature of the study.

The majority of patients in this study were referred to our centre having had their initial surgeries performed at other hospitals by general practitioners and only 6.1% of ureteric injuries were related to abdomino-pelvic surgeries performed at our centre mainly by junior doctors. Similar observation was reported by Tijani *et al.* [10] in Nigeria. High incidence of ureteric injuries among patients who had abdomino-pelvic operations performed in peripheral hospitals may be attributed to the fact that in this hospitals abdomino-pelvic surgeries were performed by general practitioners who might have limited experience.

In keeping with other studies [10,11,19], the peak age incidence of iatrogenic ureteric injuries in this study was found to be in the third decade of life indicating that this often befalls women during the reproductive periods.

In this study, females were more affected than males, an observation which is in accordance with the results of other workers [10,14,19]. The female predominance demonstrated in this study can be explained by the fact that the most common cause of iatrogenic ureteric injuries in this study was related to obstetric and gynaecological procedures in more than fifty-percent of patients.

In agreement with other studies [10,11,14,19,22], total abdominal hysterectomy (TAH) was the leading cause of ureteric injuries in this study contributing to 69.2% of cases. The ureter is most at risk for injury during gynecologic procedures (such as TAH) at the pelvic brim, beneath the infundibulopelvic ligaments, where it is crossed by the uterine artery, near the cervix [9,11]. In sub-Saharan Africa including Tanzania, with an endemic scarcity of gynaecologists, the practice of major gynaecological surgical procedures such as TAH is not limited to the specialists alone [12]. Ureteric injury may result from such practices and if not properly managed could lead to increase in morbidity and mortality [12]. Injuries may however be almost unavoidable in some situations, even in the hands of the most skilled and experienced gynaecologists. In this study, the majority of abdomino-pelvic operations including TAH were performed by general practitioners in peripheral hospitals who might have limited experience to perform major abdomino-pelvic operations such as TAH. The best defenses against ureteric injury are meticulous surgical technique as well as identification of the course of the ureter and the associated anatomic locations where injury is most likely to occur.

Patients at high risk of iatrogenic ureteric injuries include those with altered anatomy, fibrosis or direct extension of disease process, as in cases of chronic pelvic inflammatory disease, endometriosis, large fibroids (especially in the broad ligament), previous pelvic surgery, malignancy, previous irradiation and congenital abnormalities of the urogenital system [16,17]. Surgeon’s experience is also an important risk factor [18-20]. In our series, gross pelvic adhesions from previous surgeries, previous history of pelvic inflammatory diseases and massive intraoperative bleeding during operations for large pelvic tumours were reported to be risk factors for iatrogenic ureteric injuries.

In the present study, more than half of patients were operated on emergency basis which is in keeping with Tijani et al. [10] in Nigeria, but at variance with other authors [19,21] who reported that majority of patients were operated electively. In emergency situations, the surgeon typically has inadequate time to prepare the patient and to perform meticulous surgery; in the process, the surgeon may clamp or even transect a vessel along with the ureter; moreover, at odd hours of the day, competent hands may not always be available.

Ureteric injuries have been found to be more common on the left side [9,10]. This was also observed in our series, where more than half of the ureteric injuries were on the left side, but at variance with that of Oboro et al. [14] who reported that the right ureter was commonly injured. The left ureter has a greater proximity to the cervix compared to the right ureter, and is thus more liable to injury [9]. Bilateral ureteric injuries have been reported in literature to occur in 5–10% of patients [21]. In this study, bilateral injury was seen in 20.4%. These figures are comparable to that reported by other studies [6,10,22,23].

Most ureteric injuries occur in the distal most part of the ureter where it is closely related to the uterine vessels [9,11]. In the present study, more than three quarter of ureteric injuries occurred in the distal portion of the ureter, which is in agreement with other studies [10,11,22,24]. The distal portion of the ureter is not only embryologically related to the female genital organs but is also involved in diseases affecting them [9]. At the base of the cardinal ligament where it crosses the uterine artery, the ureter is just 12 mm from the vagina and as it moves towards the bladder it becomes even closer to the vagina, and this predisposes the ureter to injury during surgical procedures in the pelvis [9,11].

Ligation injury was the most common form of iatrogenic ureteric injuries reported in the operation notes by accounting for about 36.6% of cases. This is consistent with the findings of Chinakwana *et al.* [13] and most other reports in literature [10,25] but contrasts with the findings of Oboro *et al.* [14] who reported transection injury as the most common.

The management of ureteric injury depends on the time of diagnosis, patient’s condition, nature and site of ureteric injury. If the injury is recognized intra-operatively then it must be managed immediately [10,26]. In this study, the diagnosis of ureteric injury was made intra-operatively in less than twenty percent (17.1%) of the patients. However, immediately intra-operatively management of ureteric injury was not performed in any of our patients due to the fact that the majority of primary abdomino-pelvic operations were performed by general practitioners in peripheral hospitals who might have limited experience in the immediate management of ureteric injury.

Many authors have suggested that early recognition and management of ureteric injuries is associated with better outcome [21,22]. In agreement with other studies performed in developing countries [10,11,14,19], the diagnosis of ureteric injury was usually delayed at the Bugando Medical Centre in more than seventy percent of patients as majority of them were referred from peripheral hospitals. Delayed diagnosis of ureteric injury is often associated with high morbidity, uretero-vaginal fistula and the potential loss of kidney function or even mortality. The risk of impairment or loss of kidney function is increased when ureteral injury is not recognized until the post-operative period when ureteral obstruction or urinary leakage is diagnosed in an expected uneventful convalescent period [10]. In this study, missed ureteric injuries were reported in 8 (4.9%) patients and more than three quarters were found to have non-functioning kidney which necessitated nephrectomy.

Imaging is essential for diagnosis especially in cases where bilateral lesions are suspected. Ultrasound may show the eventual hydronephrosis, but this may not be obvious initially. Repeated ultrasound examinations may be necessary. Intravenous pyelogram is indispensable in delineating the degree and level of ureteric obstruction [11]. Delayed function of the involved kidney, poor function on one side, hydronephrosis, extravasation of contrast, and the finding of a urinoma are late findings that all point to ureteral injury [25]. In this study, pre-operative assessment included Ultrasound scan, Intravenous urography (IVU), Computed tomography (CT) scan, examination under anaesthesia, dye test and cystoscopy as indicated.

The objectives of the surgery for repair of iatrogenic ureteric injuries include amongst others preservation of renal function on the affected side and restoration of anatomic continuity of the urinary tract. There is a general agreement that when inadvertent injury to the ureter is detected during surgery, immediate repair is the treatment of choice [10,26]. However, when the diagnosis is delayed the optimal time for definitive treatment is subject to some controversies. Some have advocated for immediate repair while many others still prefer a delayed repair usually preceded by a period of upper tract drainage [10,17,25]. However, recent studies have reported similar results after early and delayed repair [6,17,27,28]. Early repair is associated with shorter hospital stay compared to delayed repair. In a study by Al-Awadi *et al.* [6] mean hospital stay was 4.8 days after early repair; hospital stay was 10.1 days after delayed repair. In our study, all the patients with delayed diagnosis underwent open surgical repair soon after diagnosis and stabilization. This is comparable with other studies [10,11,19,21]. Percutaneous nephrostomy has been reported to be essential when ureteric injury and obstruction is associated with infection or if the patient is not stable for definitive surgery [10,11]. In some cases nephrostomy is essential in order to preserve renal function when scarring and fibrosis at site of ureteric injury prevents immediate repair. A nephrostomy can also permit ante grade contrast studies, ureteroscopy and or ante grade ureteric catheterization [11]. In the present study, percutaneous nephrostomy was performed in only 7 patients representing 4.3% of cases. However, nephrostomy tube in this study was used only to stabilize the patients and all of these patients required additional definitive procedures.

Various reconstructive surgical options include ureteroneocystostomy, Boari flap, Psoas hitch, end to end ureteroureterostomy and trans-ureteroureterostomy [6,22,27]. Other options include ileal segment replacement, appendix interposition and auto-transplantation [10,11]. In our study, fifty-eight percent of patients underwent ureteroneocystostomy which is consistent with other studies [10,14,19]. Ureteroneocystostomy with Boari flap and Psoas hitch was performed in 16.7% of cases. In our study, nephrectomy in patients who had non-functioning kidneys was carried out in 5.6% of cases; this is similar to that reported by other authors [10].

As reported by others [10,19], surgical site infection was the most common postoperative complication in our study. High rate of surgical site infection in the present study may be attributed to contamination of the laparotomy wound during the surgical procedure.

In this study, mortality rate was 7.3% which is higher than that reported by others. [10,11]. High mortality rate in this study is attributed to delayed presentation, deranged renal function tests on admission, missed ureteric injuries and presence of postoperative complications mainly surgical site infections.

The overall median length of hospital stay was 12 days, a figure which is higher than that reported by Al-Awadi *et al*. [6]. Our overall median length of hospital stay was significantly long in patients who developed complications postoperatively. Delayed diagnosis and treatment were also associated with longer hospital stay. Prolonged length of hospitalization results in consumption of large amounts of healthcare resources such as personnel, theatre space, medications, and hospital beds.

The outcome of our management was generally good, with a 92.7% success rate and no complications that were detected during the follow up period. This may be related to the fact that the all reconstructive procedures were performed by experienced urologists. However, the follow up of patients were generally poor as more than half of patients were lost to follow up.

The potential limitation of this study is the fact that information about some patients was incomplete in view of the retrospective nature of the study and this might have introduced some bias in our findings. However, despite this limitation, the study has provided local data that can help healthcare providers in the management of patients with iatrogenic ureteric injuries. The prevention of ureteric injuries during abdomino-pelvic operations is of utmost important. Surgeon must have adequate knowledge of abdominal and pelvic anatomy especially the close relation of ureter with adjacent structures. Urologist must be consulted in the preoperative period in patients at risk for ureteral injury such as those with pelvic adhesions, pelvic radiation, pelvic malignancy, extensive surgery etc. Appropriate imaging of the urinary tract may be required in the preoperative period.

## Conclusion

Our experience in this study shows that iatrogenic ureteric injuries are still common in our environment and total abdominal hysterectomy accounts for most cases. The majority of injuries are a result of complications of abdomino-pelvic operations by general practitioners in the peripheral hospitals. Meticulous surgical technique as well as identification of the course of the ureter and associated anatomic locations where injury is most likely to occur is important to decrease the risk of ureteric injury. Early recognition and prompt repair of ureteric injuries is the key to a successful outcome. Treatment of these injuries by experienced team may minimize long-term consequences.
